# A New Medical Practice Bill

**Published:** 1906-04

**Authors:** 


A Monthly Review of Medicine and Surgery.

*EDITOR:*

WILLIAM WARREN POTTER, M. D.

All communications, whether of a literary or business nature, books for review and exchanges, should be addressed to the editor 284 FRANKLIN ST., BUFFALO, N. Y.

VOL. LXI. APRIL, 1906. NO. 9

### A New Medical Practice Bill

THE committee on public health of the assembly introduced a bill on March 22, 1906, "to regulate the practice of medicine," which repeals the law of 1893, and acts amendatory thereof, —that is to say, the law under which we are now governed.

This new bill, number 1715, proposes some radical changes in the present methods of organisation and administration, the chiefest of which we will point out. In the first place the bill abrogates the present tripartite system of medical examining boards, and creates instead a single board consisting of nine members. The appointing power remains with the regents, but nominations by the several state medical societies are not required. In other words, the regents are vested with absolute power of appointment, independently of the medical profession. A secretary is to be appointed by the regents, to hold office during their pleasure, who is to receive a salary of $4,000. a year.

The topics for examination prescribed by the bill are anatomy, physiology and hygiene, chemistry, surgery, obstetrics, gynecology, pathology and diagnosis, bacteriology, and medical jurisprudence. It will be observed that this schedule of topics eliminates practice, materia medica, and therapeutics from the list as given in the present law.

The bill under consideration is understood to be the joint product of the committee of public health of the Senate and Assembly, and is expected to silence the noisy demands for independent recognition by various sects that annually beseech and besiege the legislature. It, however, is difficult to comprehend how it is possible for one legislature to guarantee what another legislature will do or will not do in a contingency. To the uninitiated person looking on from the outside, it would appear probable that, whatever the present legislature may deem wise to enact, or no matter what it does enact, future legislatures will be obliged to meet the propositions that confront them and to deal with them as seems best at the time, irrespective of what their predecessors may have done under similar conditions.

The osteopaths are likely to be just as clamorous in the future as in the past, until they are given to understand in plain terms that theirs is not a scientific body, and no legal enactment can make it so; that if they wish to be called doctors they must take the prescribed course of study and obtain the degree in the same manner as other American citizens are obliged to do; and, finally, no matter what other commonwealths have done or may do, the Empire State will not lower its educational standards, nor grant special privileges, to any group of its citizens no matter how respectable, nor how honest their intentions.

As far as the new medical bill is concerned we can trust the legislature to gain all the light possible on the educational question in this state, and then to do the right thing—to act with equity toward the ten thousand physicians who have been commissioned by law to protect seven millions of people against the ravages of preventable disease.

---

It has recently been our privilege to sample the oil and olives produced by Mr. T. W. Chapman formerly of San Fernando, Los Angeles County, California, now residing in this city and demonstrating the good qualities of both at his business office, 46 West Huron Street. Physicians throughout the world recogonize the high medicinal and food values of strictly pure olive oil. It is worth while to know where to get the genuine at all times, and we take pleasure in calling attention to Mr. Chapman's advertisement elsewhere in this issue.

